# Social Isolation, Brain Food Cue Processing, Eating Behaviors, and Mental Health Symptoms

**DOI:** 10.1001/jamanetworkopen.2024.4855

**Published:** 2024-04-04

**Authors:** Xiaobei Zhang, Soumya Ravichandran, Gilbert C. Gee, Tien S. Dong, Hiram Beltrán-Sánchez, May C. Wang, Lisa A. Kilpatrick, Jennifer S. Labus, Allison Vaughan, Arpana Gupta

**Affiliations:** 1 Goodman-Luskin Microbiome Center, University of California, Los Angeles; 2 G. Oppenheimer Center for Neurobiology of Stress and Resilience, University of California, Los Angeles; 3 Vatche and Tamar Manoukian Division of Digestive Diseases, University of California, Los Angeles; 4 School of Medicine, University of California, San Diego, La Jolla, California; 5 Department of Community Health Sciences, Fielding School of Public Health, University of California, Los Angeles; 6 California Center for Population Research, University of California, Los Angeles; 7 David Geffen School of Medicine at the University of California, Los Angeles

## Abstract

**Importance:**

Perceived social isolation is associated with negative health outcomes, including increased risk for altered eating behaviors, obesity, and psychological symptoms. However, the underlying neural mechanisms of these pathways are unknown.

**Objective:**

To investigate the association of perceived social isolation with brain reactivity to food cues, altered eating behaviors, obesity, and mental health symptoms.

**Design, Setting, and Participants:**

This cross-sectional, single-center study recruited healthy, premenopausal female participants from the Los Angeles, California, community from September 7, 2021, through February 27, 2023.

**Exposure:**

Participants underwent functional magnetic resonance imaging while performing a food cue viewing task.

**Main Outcomes and Measures:**

The main outcomes included brain reactivity to food cues, body composition, self-reported eating behaviors (food cravings, reward-based eating, food addiction, and maladaptive eating behaviors), and mental health symptoms (anxiety, depression, positive and negative affect, and psychological resilience).

**Results:**

The study included 93 participants (mean [SD] age, 25.38 [7.07] years). Participants with higher perceived social isolation reported higher fat mass percentage, lower diet quality, increased maladaptive eating behaviors (cravings, reward-based eating, uncontrolled eating, and food addiction), and poor mental health (anxiety, depression, and psychological resilience). In whole-brain comparisons, the higher social isolation group showed altered brain reactivity to food cues in regions of the default mode, executive control, and visual attention networks. Isolation-related neural changes in response to sweet foods correlated with various altered eating behaviors and psychological symptoms. These altered brain responses mediated the connection between social isolation and maladaptive eating behaviors (β for indirect effect, 0.111; 95% CI, 0.013-0.210; *P* = .03), increased body fat composition (β, −0.141; 95% CI, −0.260 to −0.021; *P* = .02), and diminished positive affect (β, −0.089; 95% CI, −0.188 to 0.011; *P* = .09).

**Conclusions and Relevance:**

These findings suggest that social isolation is associated with altered neural reactivity to food cues within specific brain regions responsible for processing internal appetite-related states and compromised executive control and attentional bias and motivation toward external food cues. These neural responses toward specific foods were associated with an increased risk for higher body fat composition, worsened maladaptive eating behaviors, and compromised mental health. These findings underscore the need for holistic mind-body–directed interventions that may mitigate the adverse health consequences of social isolation.

## Key Points

**Question:**

Is perceived social isolation associated with brain reactivity to food cues, obesity, and psychological symptoms?

**Findings:**

In this cross-sectional study of 93 healthy, premenopausal female participants, social isolation was associated with altered brain processing of food cues in the default mode, executive control, and visual attention networks. These neural changes (especially to sweet foods) showed an association among social isolation, eating behaviors, and psychological symptoms.

**Meaning:**

These findings indicate that increased loneliness may be linked to brain patterns that highlight difficulties in motivation, control, and processing of internal states in response to foods and increased alterations in eating behaviors, obesity, and psychological symptoms, suggesting future targets for obesity treatments.

## Teaser

This cross-sectional study investigates the association of perceived social isolation with brain reactivity to food cues, eating behaviors, obesity, and mental health in women.

## Introduction

Perceived social isolation, often referred to as loneliness, reflects one’s subjective appraisal of their social relationships and community support.^1^ Loneliness is an entirely subjective state and doesn’t necessarily rely on the quantity of one’s social relations.^2^ Despite its connection to objective social isolation (social disconnectedness), characterized by having limited or infrequent social contact, loneliness is distinct and may impact health.^3,4,5^ Loneliness has been found to impact well-being^6,7,8^ and to be a risk factor for early mortality and chronic conditions, such as cardiovascular disease and atherosclerosis.^7,8^ The aftermath of COVID-19 highlighted a multitude of health concerns that emerged in conjunction with social isolation, including increased obesity, unhealthy eating behaviors, physical and psychological disorders, and cognitive decline, underscoring the need to better understand the underlying physiology.^1,9,10,11,12^ Yet, few studies have examined the biological mechanisms linking perceived social isolation to health outcomes.

Perceived social isolation is associated with brain alterations in the default mode network (DMN), executive control network (ECN), visual attention network (VAN), and reward network (RN).^13,14,15,16,17,18,19,20,21,22^ Alterations in the DMN have been shown to increase the propensity of individuals experiencing loneliness to resort to self-rumination and internal generation of thoughts as a compensatory mechanism for a lack of social interactions.^17,23^ Increased connectivity in areas of attentional processing, particularly in visual cortices and cognitive control areas, suggests that individuals experiencing loneliness may continually monitor their surroundings for perceived social threats.^16,19,20,23^ This implicit hypervigilance combined with an increased sensitivity to negative social stimuli could imply that loneliness primes individuals to display a magnified instinct for self-preservation and impaired social perception skills.^21,22^

Loneliness also may increase the risk for obesity and worsened eating behaviors and disorders,^9,12,24,25^ specifically with associations to increased sugar consumption and cravings for sugary beverages.^26,27^ Sedentary behavior, often accompanied by social isolation, has been found to contribute to the development of depression, anxiety, suicidal behavior, personality disorders, and psychosis.^2,10,28^ Individuals experiencing loneliness present heightened attentional bias and affective processing to negative social cues, along with diminished affective responsiveness to positive social interactions, within the VAN and affective processing network.^29,30,31^ These brain alterations may underlie the intricate connection between social isolation and mental health. When mental health disorders affect an individual’s capacity to self-regulate emotional well-being, binge eating behaviors may emerge as a coping mechanism to combat the negative affect perceived during prolonged periods of social isolation.^25,32^ Individuals experiencing loneliness also display increased activation in areas of the RN (ie, ventral striatum, insula, nucleus accumbens).^13^ These RN alterations result in intense cravings also seen in individuals with drug and food addiction,^14^ suggesting that social isolation may increase processing of reward-based regions by altering midbrain dopaminergic neurons, thereby inducing a craving for social reconnection and engagement.^13,14,33^ Similar modulations in the brain’s RN when an individual is in any deprived state (ie, hunger, loneliness, drug withdrawal) highlight that social connections may affect pathways related to stress and coping.^34,35^

Emerging evidence points to the potential role of a “lonely brain” that may contribute to obesity, altered eating behaviors, and associated psychological symptoms. Therefore, our study aims to elucidate the general neural mechanisms linking loneliness to alterations in neural responses to food cue processing to test the following 3 hypotheses (eFigure 1 in Supplement 1): First, loneliness is associated with increased activation in the DMN, VAN, ECN, and RN regions of the brain when viewing food cues compared with nonfood cues. Second, loneliness-associated brain reactivity is correlated with increased obesity measures, altered eating behaviors, and worsened mental health. Third, sweet food–related neural alterations, compared with savory food, may show a stronger association with maladaptive eating behaviors and mental health outcomes, given sugary foods’ highly rewarding nature and analgesic effect that may alleviate the “social pain” associated with social exclusion,^36,37,38^ thereby contributing to the complex interplay between loneliness and health outcomes.^38,39^

## Methods

### Participants

This cross-sectional study included healthy, premenopausal female participants who were recruited from the Los Angeles, California, community through advertisements from September 7, 2021, through February 27, 2023. Detailed recruitment and exclusion criteria and procedures are provided in the eMethods in Supplement 1. All procedures complied with institutional guidelines and were approved by the institutional review board of the University of California, Los Angeles Office of Protection for Research Subjects. All participants provided written informed consent. The study followed the Strengthening the Reporting of Observational Studies in Epidemiology (STROBE) reporting guideline.

Participant data included body mass index (BMI), body composition, diet style and quality,^40,41^ self-reported race and ethnicity, age, marital status, and socioeconomic status.^42^ Data on race and ethnicity were collected to enhance transparency and provide preliminary data for future research on the effects of isolation on brain health in specific groups or as a comparison baseline with other races and ethnicities. In addition, all participants underwent a bioelectrical impedance analysis using a body composition analyzer to evaluate body composition through electrical tissue conductivity, providing estimates of body fat and lean body mass percentages. Multimodal data, including functional magnetic resonance imaging (fMRI) and clinical and behavioral measures, were also collected.

### Clinical and Behavioral Assessments

Perceived social isolation was assessed using the validated Perceived Isolation Scale.^43^ This scale measures the frequency of emotional and instrumental support from family, friends, and partners as well as feelings of companionship, exclusion, and isolation.^43^ The coding and scoring methodology for this scale has been described in a previous study,^5^ which involved standardization of each individual item followed by the calculation of a mean score across all scale items.^43^ Scores were dichotomized to categorize participants into a high perceived isolation group (higher than the mean) and a low perceived isolation group (lower than the mean) based on the sample mean as described in a prior study.^5^

Other clinical and behavioral measures were examined both individually and by categorizing them into the following composite groups: (1) body measurements (BMI, fat mass percentage, and lean body mass percentage), (2) eating behaviors (food cravings,^44^ reward-based eating,^45^ maladaptive eating behaviors,^46^ and food addiction symptoms, with higher scores indicating more of such eating behaviors),^47^ and (3) mental health variables (psychological resilience,^48^ anxiety symptoms,^49^ depression symptoms,^49^ positive affect, and negative affect).^50^ Details regarding the measurements and questionnaires are provided in the eMethods in Supplement 1.

### Baseline Characteristics

Baseline demographic and clinical characteristics were compared between the high and low perceived isolation groups by Student *t* test for continuous variables and χ^2^ test for categorical variables using R, version 4.2.3 (R Project for Statistical Computing).^51^ Effect sizes for *t* tests are Cohen *d* values, and effect sizes for χ^2^ tests are reported as the standardized mean difference. For all tests of significance, *P* < .05 was considered statistically significant, with no adjustment for multiple comparisons.

### Food Cue Task MRI Acquisition, Processing, and Analyses

Brain data were acquired using a 3.0-T Prisma MRI scanner (Siemens). Acquisition details are provided in the eMethods in Supplement 1. Participants were asked to fast for approximately 6 hours prior to scanning, which was confirmed by the study coordinator (A.V.). Participants performed a food cue task while in the scanner to assess neural responses to various foods (savory, sweet) and nonfood (pixelated control) images. Details of the food cue task have been published in a previous study^52^ and are detailed in the eMethods in Supplement 1.

Neuroimaging data were processed using the fMRI Expert Analysis Tool, version 6.0 included in the FMRIB Software Library (FSL).^53^ Details regarding preprocessing are provided in the eMethods in Supplement 1.

### Perceived Isolation–Related Brain Reactivity to Food Cues

To determine perceived isolation–related differences in whole-brain food cue reactivity, we specified the following contrasts: (1) food vs nonfood, (2) sweet food vs nonfood, and (3) savory food vs nonfood. The corresponding reversed contrasts for the above were also specified. For each participant, 6 contrast maps were created in the first-level analysis and then entered into the random-effects group-level analyses using FSL’s Local Analysis of Mixed Effects in a whole-brain analysis with outlier deweighting. Independent *t* tests (high perceived isolation vs low perceived isolation) with age as a covariate were performed using the FSL fMRI Expert Analysis Tool. All statistical maps were family-wise error cluster corrected for multiple comparisons (cluster height threshold: *z* > 2.3; cluster significance, *P* < .05).

Food cue regions of interest (ROIs) were created from the significant clusters in the whole-brain contrasts (described above). Brain signal change (β-values from the first-level statistical models) were extracted for each participant separately from each ROI. The food cue reactivity using brain signal change extracted from the food cue ROIs were entered into the association and mediation analyses (described below).

### Perceived Isolation–Related Brain Food Cure Reactivity and Clinical and Behavioral Assessments

Multiple linear regression analyses were conducted to examine the associations between loneliness-related brain food cue reactivity within each ROI and individual clinical and behavioral measures that exhibited significant differences between the high perceived isolation and low perceived isolation groups while adjusting for age. We also calculated composite scores for each category using the individual measures that showed significant differences between the 2 groups. We summed all standardized measures within each composite category after reversing any reverse-coded measures (ie, psychological resilience, positive affect). The composite categories included body measurement, eating behaviors, and mental health, which were used in the mediation analyses described below.

### Mediation Analyses

Mediation analyses using structural equation modeling (SEM) assessed the mediating (indirect) effect of brain food cue reactivity on the association between perceived isolation and outcomes of interest (eg, various individual measures that differed significantly between high and low perceived isolation groups). Perceived isolation (high vs low coded as 1 vs −1) was entered into the model as a predictor variable. Mediation analyses were performed with the lavaan package in R^51^ when significant differences were observed between the high and low perceived isolation groups in the initial analyses. Brain reactivity to different types of food cues was entered as the mediator.

We grouped the aforementioned individual outcomes into 3 composite categories as latent variables (ie, body measurement, eating behaviors, mental health). We examined the indirect effect of brain reactivity to different food cues on the association between perceived isolation and these composite categorical outcomes. All mediation models were adjusted for age, and standardized β for indirect effect was reported. The first indicator of each latent variable was fixed to 1, and maximum likelihood robust estimation was used to fit all models. The significance level was set at *P* < .05 for all SEM statistical significance testing. We additionally indicated results at *P* < .10, considering the preliminary nature of our study, to capture potentially significant findings that require further investigation.

## Results

### Participant Characteristics

The 93 female participants were aged 18 to 50 years (mean [SD], 25.38 [7.07] years), including 38 who self-reported as Filipino (41%) and 55 as Mexican (59%) (Table 1). Compared with the low perceived isolation group (n = 54), the high perceived isolation group (n = 39) showed significantly higher fat mass percentage (mean [SD], 29.70% [5.63%] vs 32.63% [6.80%]; *P* = .04), lower diet quality (mean [SD] Healthy Eating Index, 66.60 [10.26] vs 61.95 [10.51]; *P* = .04), increased maladaptive eating behaviors (mean [SD] scores: food cravings, 31.26 [11.84] vs 37.16 [13.21] [*P* = .03]; reward-based eating, 0.65 [0.73] vs 1.26 [0.93] [*P* = .001]; uncontrolled eating, 1.77 [0.53] vs 2.90 [3.17] [*P* = .04]; food addiction, 1.13 [1.17] vs 1.87 [1.78] [*P* = .03]), and poorer mental health (mean [SD] scores: psychological resilience, 71.71 [14.00] vs 63.37 [12.01] [*P* = .003]; anxiety, 6.90 [4.06] vs 9.40 [4.46] [*P* = .008]; depression, 3.73 [3.49] vs 5.27 [3.33] [*P* = .04]; positive affect, 30.15 [9.35] vs 26.18 [8.83] [*P* = .04]). Detailed definitions for each index are provided in the eMethods and eTable in Supplement 1.

**Table 1.** Participant Characteristics

Characteristic	Mean (SD) [range]		*P* value	Effect size
	High perceived isolation (n = 39)	Low perceived isolation (n = 54)		
Age, y	25.92 (7.83) [18.00-50.00]	24.98 (6.51) [18.00-42.00]	.54	0.13
Marital status, No. (%)				
Married	33 (85)	50 (93)	.17	0.29
Not married	6 (15)	4 (7)		
Race and ethnicity, No. (%)				
Filipino	18 (46)	20 (37)	.57	0.12
Mexican	21 (54)	34 (63)		
Diet, No. (%)				
Standard American diet	24 (62)	36 (67)	.61	0.11
Nonstandard American diet	15 (38)	18 (33)		
**Indexes** ^a^				
Diet quality, Healthy Eating Index^b^	61.95 (10.51) [37.75-79.28]	66.60 (10.26) [44.21-85.47]	.04^c^	−0.45
Body measurements				
BMI	27.07 (5.54) [20.20-40.35]	25.17 (4.99) [18.10-42.00]	.11	0.36
BIA fat mass, %	32.63 (6.80) [23.20-46.90]	29.70 (5.63) [20.70-43.70]	.04^c^	0.47
BIA lean body mass, %	67.20 (7.14) [51.10-76.80]	70.12 (5.77) [56.30-79.30]	.05^d^	−0.45
Eating behaviors				
Food cravings	37.16 (13.21) [16-68]	31.26 (11.84) [15-60]	.03^c^	0.47
Reward-based eating drive	1.26 (0.93) [0-3.67]	0.65 (0.73) [0-2.78]	.001^e^	0.73
Three-facet eating			.55	0.13
Cognitive restraint^f^	3.41 (3.34) [1.50-18.00]	3.00 (3.03) [1.33-18.00]		
Uncontrolled eating^g^	2.90 (3.17) [1.00-18.00]	1.77 (0.53) [1.00-2.89]	.04^c^	0.49
Emotional eating^h^	2.68 (2.28) [1.00-15.60]	2.14 (2.09) [1.00-13.20]	.25	0.25
Food addiction symptoms	1.87 (1.78) [0-7.00]	1.13 (1.17) [0-5.00]	.03^c^	0.49
Mental health				
Psychological resilience^i^	63.37 (12.01) [36.00-87.00]	71.71 (14.00) [30.00-100.00]	.003^e^	−0.64
Anxiety^j^	9.40 (4.46) [0-19.00]	6.90 (4.06) [0-15.00]	.008^e^	0.58
Depression^j^	5.27 (3.33) [0-13.00]	3.73 (3.49) [0-14.00]	.04^c^	0.45
Positive affect^k^	26.18 (8.83) [11.00-47.00]	30.15 (9.35) [10.00-50.00]	.04^c^	−0.44
Negative affect^k^	14.39 (3.94) [10.00-29.00]	13 (3.05) [10.00-21.00]	.07^d^	0.39

Abbreviations: BIA, bioelectrical impedance analysis; BMI, body mass index (weight in kilograms divided by height in meters squared).

^a^ Higher scores for each index indicate a greater presence of each measurement. Detailed definitions for each index can be found in the eMethods and eTable in Supplement 1.

^b^ Healthy Eating Index score ranges from 0 to 100, with higher scores indicating better dietary quality.

^c^ Statistically significant at *P* < .05.

^d^ Statistically significant at *P* < .10.

^e^ Statistically significant at *P* < .01.

^f^ Maximum possible score is 21.

^g^ Maximum possible score is 16.

^h^ Maximum possible score is 14.

^i^ As measured using the Connor-Davidson Resilience Scale, with each item self-rated on a scale of 0 to 4, with higher overall score indicating greater resilience.

^j^ As measured using the Hospital Anxiety and Depression Scale, with each item scored on a scale of 0 to 3, with higher scores indicating greater anxiety or depression in the past week.

^k^ As measured using the Positive and Negative Affect Schedule.

### Perceived Isolation–Related Brain Reactivity to Food Cues

**Figure 1.**
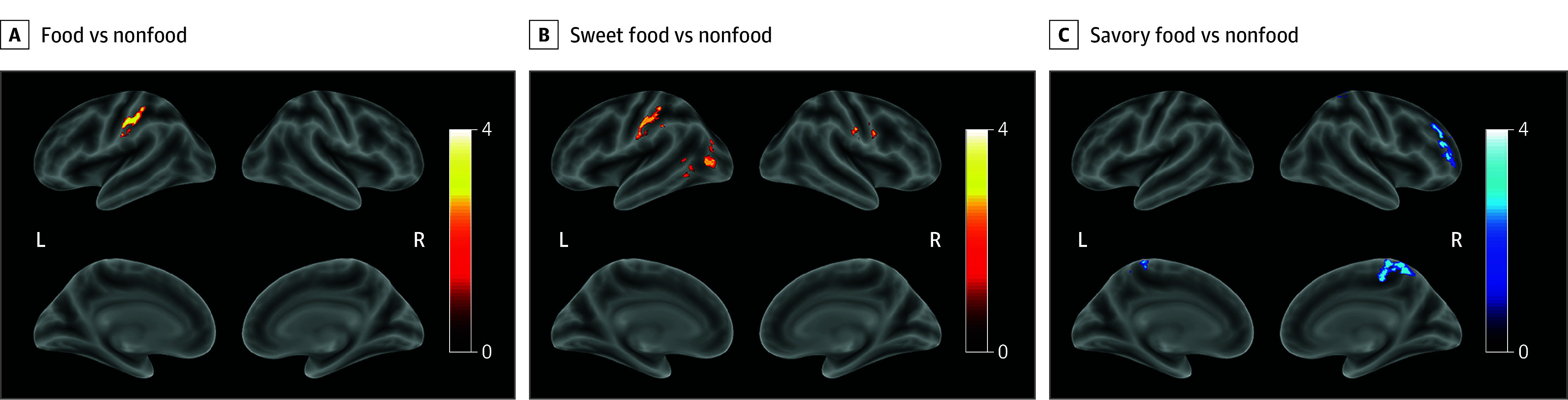
Whole-Brain Comparisons Between High and Low Perceived Isolation Groups Warm-colored areas (red) indicate greater reactivity in the high perceived isolation group than in the low perceived isolation group. Cold-colored areas (blue) indicate greater reactivity in the low perceived isolation group than in the high perceived isolation group. Comparison results are adjusted for age. Family-wise error cluster-level correction: *z* score greater than 2.3 (*P* < .05). Clusters are listed in Table 2. The color bar represents the *z* score thresholding at 2.3, with warmer (lighter yellow) and colder (lighter blue) colors indicating higher *z* scores. L indicates left hemisphere; R, right hemisphere.

In whole-brain comparisons, when viewing foods vs nonfoods, the high perceived isolation group had greater food cue reactivity than the low perceived isolation group in the inferior parietal lobule (IPL) (Figure 1A). When viewing sweet foods vs nonfoods, the high perceived isolation group had greater food cue reactivity than the low perceived isolation group in the IPL, inferior frontal gyrus, and lateral occipital cortex (Figure 1B; Table 2). When viewing savory foods vs nonfoods, the high perceived isolation group had less food cue reactivity than the low perceived isolation group in the central precuneus and dorsolateral prefrontal cortex (dlPFC) (Figure 1C; Table 2).

**Table 2.** Significant Clusters From Whole-Brain Analysis of Food Cue Reactivity Comparing High and Low Perceived Isolation Groups

Contrast and cluster region	Peak voxel coordinates, MNI space			Maximum *z* score^a^
	x-Axis	y-Axis	z-Axis	
**Food vs nonfood: high > low perceived isolation**				
L, inferior parietal lobule	−60	−20	46	4.07
**Sweet food vs nonfood: high > low perceived isolation**				
L, inferior parietal lobule	−62	−18	42	4.58
R, inferior frontal gyrus	60	18	34	3.62
L, lateral occipital cortex, inferior division	−46	−82	4	3.35
**Savory food vs nonfood: low > high perceived isolation**				
L, central precuneus	−6	−46	60	4.23
R, dlPFC	40	44	24	3.92

Abbreviations: dlPFC, dorsolateral prefrontal cortex; L, left hemisphere; MNI, Montreal Neurological Institute (measured in millimeters); R, right hemisphere.

^a^ Family-wise error cluster-level correction: *z* score greater than 2.3 (*P* < .05). Comparison results are controlled for age.

### Associations Between Perceived Isolation–Related Brain Food Cue Reactivity and Clinical and Behavioral Assessments

The associations between perceived isolation–related brain food cue (food, sweet, and savory food) reactivity and clinical and behavioral assessments are shown in Table 3. Brain reactivity to all foods, including sweet foods, was associated maladaptive eating behaviors (food vs nonfood: standardized estimate, 0.327; 95% CI, 0.320-0.335; sweet food vs nonfood: standardized estimate, 0.315; 95% CI, 0.302-0.327), and mental health outcomes (food vs nonfood: standardized estimate, 0.264; 95% CI, 0.257-0.272; sweet food vs nonfood: standardized estimate, 0.229; 95% CI, 0.217-0.242). No associations were observed for savory foods.

**Table 3.** Associations Between Perceived Isolation–Related Brain Food Cure Reactivity and Clinical and Behavioral Assessments

Parameter	Food vs nonfood		Sweet food vs nonfood		Savory food vs nonfood	
	Standardized estimate (95% CI)	*P* value	Standardized estimate (95% CI)	*P* value	Standardized estimate (95% CI)	*P* value
BIA fat mass, %	0.004 (−0.013 to 0.020)	.97	−0.112 (−0.139 to 0.086)	.30	−0.011 (−0.062 to 0.041)	.92
Eating behaviors (total combined)	0.327 (0.320 to 0.335)	.002^a^	0.315 (0.302 to 0.327)	.003^a^	−0.084 (−0.108 to −0.061)	.44
Food cravings	0.282 (0.251 to 0.314)	.008^a^	0.271 (0.217 to 0.325)	.01^b^	0.006 (−0.096 to 0.108)	.96
Reward-based eating drive	0.377 (0.374 to 0.379)	<.001^c^	0.361 (0.357 to 0.364)	<.001^c^	−0.097 (−0.104 to −0.090)	.36
Three-facet eating: uncontrolled eating	0.036 (0.030 to 0.041)	.74	0.104 (0.095 to 0.114)	.32	−0.125 (−0.141 to −0.108)	.23
Food addiction symptoms	0.231 (0.227 to 0.234)	.02^b^	0.198 (0.192 to 0.204)	.06^d^	−0.055 (−0.066 to −0.043)	.60
Mental health	0.264 (0.257 to 0.272)	.01^b^	0.229 (0.217 to 0.242)	.03^b^	−0.091 (−0.114 to −0.067)	.40
Resilience^e^	−0.171 (−0.206 to −0.135)	.11	−0.184 (−0.244 to −0.124)	.09^d^	0.089 (−0.022 to 0.200)	.40
Anxiety	0.256 (0.245 to 0.267)	.01^b^	0.165 (0.146 to 0.184)	.12	−0.086 (−0.120 to −0.051)	.41
Depression	0.166 (0.157 to 0.175)	.13	0.146 (0.131 to 0.162)	.18	−0.139 (−0.167 to −0.111)	.19
Positive affect^e^	−0.177 (−0.201 to −0.154)	.10^d^	−0.155 (−0.196 to −0.114)	.15	−0.073 (−0.149 to 0.003)	.49

Abbreviation: BIA, bioelectrical impedance analysis.

^a^ Statistically significant at *P* < .01.

^b^ Statistically significant at *P* < .05.

^c^ Statistically significant at *P* < .001.

^d^ Statistically significant at *P* < .10.

^e^ Reverse coded.

### Mediation Analyses

**Figure 2.**
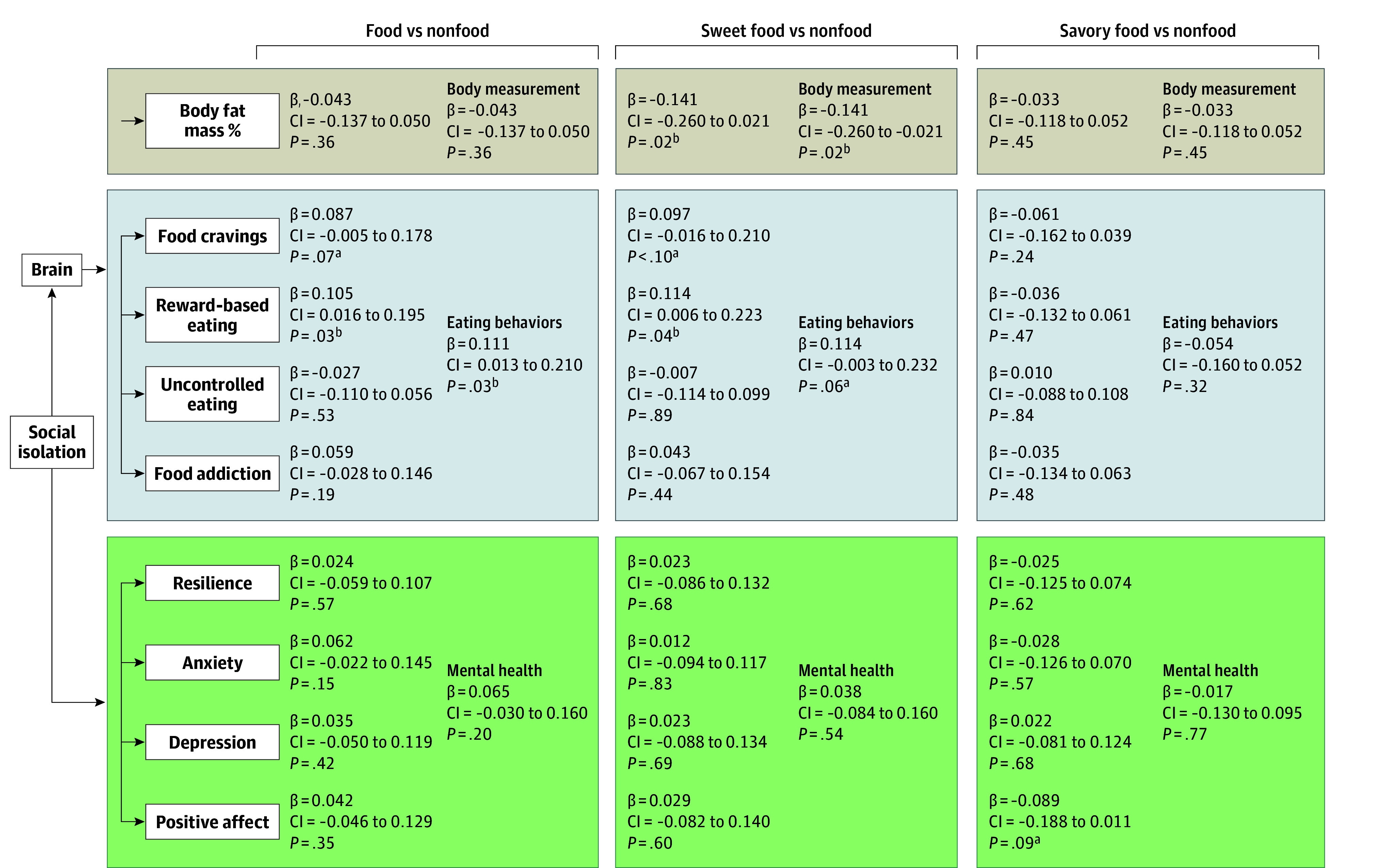
Mediation Models on Brain Reactivity to Food Cues and Association With Perceived Isolation and Body Measurement, Eating Behaviors, and Mental Health β Indicates indirect effect. All CIs are 95% CIs. ^a^Statistically significant at *P* < .10. ^b^Statistically significant at *P* < .05.

The results of all the mediation SEMs are shown in Figure 2. When viewing food vs nonfood, brain reactivity mediated the association between perceived isolation and food cravings (standardized β for indirect effect, 0.087; 95% CI, −0.005 to 0.178; *P* = .07), reward-based eating (β for indirect effect, 0.105; 95% CI, 0.016-0.195; *P* = .03), and overall maladaptive eating behaviors (β for indirect effect, 0.111; 95% CI, 0.013-0.210; *P* = .03). When viewing sweet food vs nonfood, brain reactivity mediated the association between perceived isolation and body fat mass percentage (β for indirect effect, −0.141; 95% CI, −0.260 to −0.021; *P* = .02), food cravings (β for indirect effect, 0.097; 95% CI, −0.016 to 0.210; *P* < .10), reward-based eating (β for indirect effect, 0.114; 95% CI, 0.006 to 0.223; *P* = .04), and overall maladaptive eating behaviors (β for indirect effect, 0.114; 95% CI, −0.003 to 0.232; *P* = .06). When viewing savory food vs nonfood, brain reactivity mediated the association between perceived isolation and positive affect (β for indirect effect, −0.089; 95% CI, −0.188 to 0.011; *P* = .09). Significant standardized path coefficients among brain, clinical and behavioral measures, and perceived isolation for all mediation models are shown in eFigure 2 in Supplement 1.

## Discussion

This study elucidates the neural mechanisms linking perceived social isolation and loneliness to obesity, eating behaviors, and mental health symptoms. Individuals who were lonely exhibited increased body fat composition and more maladaptive eating behaviors, alongside heightened susceptibility to psychological symptoms.

Loneliness was associated with increased brain reactivity to food cues in regions within the DMN (eg, IPL) and VAN (eg, occipital cortex) and reduced activity in the ECN (dlPFC). These neural alterations reflected an imbalance in sensitivity to internal appetite-related states and external food cues coupled with compromised executive control. These brain alterations may be a key link between social isolation and various outcomes, extending beyond eating behaviors to encompass overall mental health symptoms.

### Perceived Social Isolation and Loneliness Association With Brain Food Cue Processing

When viewing images of food, individuals who reported more perceived social isolation exhibited increased brain reactivity in the IPL, a key region within the DMN.^54,55^ The DMN is central in social behaviors, including self-relevant mentalizing, interoception, introspective awareness, and the regulation of emotional processes.^55,56,57,58^ Consistent findings have identified changes within the DMN in individuals experiencing loneliness, including shifts in structural morphology, microstructure, intrinsic functional connectivity, and task-based activation.^18,31,59,60^

Loneliness is linked to emotional processing toward external socioaffective cues and internal self-generated thoughts and cognitions.^61^ The heightened response to food cues within the DMN in individuals experiencing loneliness may reflect their increased engagement in processing internal states, such as appetite, interpretation of gut signals, or food-related cognition.^62^ The IPL is a region related to attention, multisensory integration, visual processing, and motivation.^63,64,65^ Consequently, the altered brain reactivity observed in individuals experiencing loneliness may reflect an increased salience or appeal toward food cues and elevated attentional and sensory processing and motivation directed toward these cues. This heightened internal state sensitivity (eg, appetite, hunger), coupled with an elevated external attention, sensory processing, and motivation toward food cues, potentially contributes to maladaptive eating behaviors, including reward-based eating and food addiction, as observed in our study.

Individuals experiencing loneliness exhibited heightened brain reactivity in the IPL (DMN) as well as regions within the VAN (inferior frontal gyrus and lateral occipital cortex) in response to sweet foods, indicating increased sensitivity to both internal states and external cue–induced reactivity.^66,67,68^ When viewing savory foods, individuals experiencing loneliness displayed decreased brain reactivity in the central precuneus and the dlPFC (part of the ECN).^69,70,71,72,73^ This finding aligns with previous research pointing to compromised inhibitory control in individuals experiencing loneliness.^11,21,74,75^ The precuneus plays a dual role and is considered part of the DMN and ECN.^70,76^ Importantly, it maintains connectivity with critical frontoparietal nodes responsible for executive functions during both rest and tasks involving executive control.^77,78^ Structural alterations have been observed in the dlPFC in individuals reporting higher levels of loneliness, and its functional connectivity has been shown to predict loneliness.^69,79^ Overall, these findings imply that loneliness may reshape the brain’s processing of food cues by heightening individuals’ sensitivity to appetite-related internal states, increasing the salience or motivation in response to external food cues, all while potentially compromising executive control.

In examining the neural links between social isolation and brain reactivity to food cues, sweet foods appear to exert a more pronounced and overarching influence compared with savory foods. According to social baseline theory, individuals lacking strong social connections may exhibit heightened vigilance for potential threats and increased reactivity, necessitating greater neural metabolic resources for adaptive functioning.^80^ It is possible that socially isolated individuals may experience stronger cravings and consumption of foods and beverages that rapidly raise blood glucose levels.^26,81^ Sweet food is also highly rewarding, with an analgesic effect that can reduce the social pain associated with social exclusion.^36,37,38^ Prior studies found that loneliness was associated with increased sugar consumption and heightened cravings for sugar-sweetened beverages.^26,27^ The neural responses to sweet food cues in the context of social isolation involves regions (IPL and inferior frontal gyrus) associated with the social brain and partly overlaps with the DMN, which is responsible for understanding, processing, and responding to social information and interactions.^82,83^

### Neural Mechanisms Linking Social Isolation to Eating Behaviors and Mental Health

Reactivity to food cues, especially sweet foods, in socially isolated individuals is associated with an exacerbation of maladaptive eating behaviors, including cravings, heightened reward-based eating drive, and symptoms of food addiction. This brain reactivity serves as a mediator, bridging the connection between social isolation and eating behaviors, as well as an increase in body fat percentage. Previous studies have also linked social isolation and the DMN and VAN to eating disorders, compulsive eating, unhealthy eating habits,^12,24,25^ and an increased risk for addictive behaviors and obesity.^84,85,86^

Food cue reactivity among socially isolated individuals was associated with worsened mental health, including increased anxiety and reduced positive affect and psychological resilience. This brain reactivity also served as a mediator, connecting social isolation with decreased positive affect. These findings underscore the psychiatric relevance of DMN functionality, also found to be altered in individuals with autism, schizophrenia, attention-deficit/hyperactivity disorder, Alzheimer disease, depression, and anxiety.^87,88,89,90,91,92^

Given the interconnection among all the factors examined, it is essential to evaluate the degree-dependent and causal effects of social isolation by assessing whether the extent or duration of social isolation might influence mental health susceptibility differently. Future research could consider longitudinal studies or experimental designs that adjust for confounding factors (eg, depression) and account for other loneliness-related sedentary behaviors that may contribute to obesity (eg, sleep disturbances, dysregulated immune functioning). This approach may provide a deeper exploration of the nuanced associations between loneliness and health, building on the patterns identified in this study. Adopting systemic approaches that integrate data from the gut microbiome and peripheral inflammatory markers can offer further insight into the consequences of social isolation.^93,94^ Due to the multiple testing in our study, the presence of chance findings cannot be ruled out; however, we observed consistent patterns across outcomes. Future replication of our findings may validate and build upon the insights gained in this study.

### Limitations

This study has some limitations. Because of the cross-sectional design causality cannot be inferred. The sample size is relatively small and limited to females only, and caution must be taken in interpreting the findings. Further research is needed to explore our findings in larger, more diverse populations and through longitudinal studies in order to better understand causality.

## Conclusions

This cross-sectional study highlights that social isolation is associated with altered brain processing of food cues in regions associated with the DMV, VAN, and ECN. This association is marked by a heightened sensitivity to internal appetite-related states and the external salience of food cues, along with compromised executive control. These brain alterations play a crucial role in mediating the association between social isolation and obesity, altered eating behaviors, and worse mental health outcomes. To nourish or feed the lonely brain, holistic interventions that target both body and mind for overall lifestyle improvement may offer the most effective means of mitigating the complex adverse effects of social isolation. For example, normalizing altered brain networks through mobile interventions, such as journaling and meditation, have shown improvements in self-compassion, stress reduction, and lower BMI.^95^ Similarly, exercise and diet interventions that target the brain and gut may correlate with fat loss, reduced hunger, increased psychological resilience, and improved mood.^96,97^

## Notes

Supplement 1. **eFigure 1.** Graphical Abstract
eMethods.
**eTable.** Diet Checklist Categories **eFigure 2.** Mediation Models on Brain Responses to Food Cues Associated With Perceived Isolation and Body Measurement, Eating Behaviors, and Mental Health Factors With Path Coefficients
eReferences

Supplement 2.
Data Sharing Statement
