# Supplementary material for: Social Isolation, Brain Food Cue Processing, Eating Behaviors, and Mental Health Symptoms
Source: JAMA Netw Open. 2024 Apr 4;7(4):e244855. doi: 10.1001/jamanetworkopen.2024.4855 (PMC11192185; doi:10.1001/jamanetworkopen.2024.4855)
Supplement: Supplement 1. — eFigure 1. Graphical Abstract eMethods. eTable. Diet Checklist Categories eFigure 2. Mediation Models on Brain Responses to Food Cues Associated With Perceived Isolation and Body Measurement, Eating Behaviors, and Mental Health Factors With Path Coefficients eReferences [file jamanetwopen-e244855-s001.pdf]

Supplemental Online Content

Zhang X, Ravichandran S, Gee GC, et al. Social isolation, food cue processing, eating behaviors, and mental health symptoms. *JAMA Netw Open*. 2024;7(4):e244855. doi:10.1001/jamanetworkopen.2024.4855

eFigure 1. Graphical Abstract

eMethods.

eTable. Diet Checklist Categories

eFigure 2. Mediation Models on Brain Responses to Food Cues Associated With Perceived Isolation and Body Measurement, Eating Behaviors, and Mental Health Factors With Path Coefficients

eReferences

This supplemental material has been provided by the authors to give readers additional information about their work.

© 2024 Zhang X et al. *JAMA Network Open.*

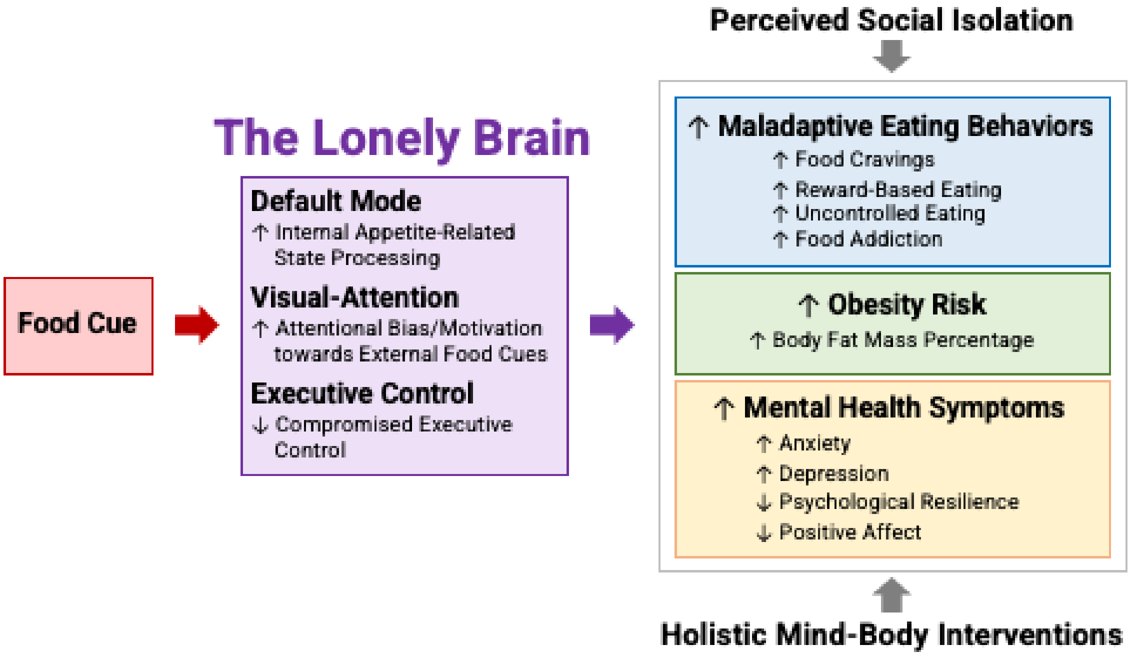

eFigure 1. Graphical Abstract.
Figure Legend: Perceived social isolation was associated with altered neural reactivity to food cues (especially to sweet foods) within the default mode, executive control, and visual-attention networks. These neural changes were responsible for processing internal appetite-related states, compromised executive control, and attentional bias and motivation towards external food cues. Neural responses towards specific foods leads to increased risk for higher body fat composition, worsened maladaptive eating behaviors, and compromised mental health. These findings underscore the need for holistic mind-body directed interventions that consider the intricate interplay of these factors in effectively mitigating the adverse consequences of social isolation.

eMethods

Participant recruitment and exclusion criteria: Women who were either peri-menopausal or post-menopausal, as determined by their self-reported last menstrual cycle, were not included in the study. The enrolled women underwent scanning during the follicular phase of their menstrual cycle. Individuals with major medical or neurological conditions, current or past psychiatric illnesses, comorbidities like vascular disease or diabetes, a history of weight loss or abdominal surgeries, substance use disorders, tobacco dependence (smoking half a pack or more daily), or those with metal implants were excluded. Additionally, individuals who used medications that could interfere with the central nervous system, regularly took analgesic medications, were pregnant or breastfeeding, or engaged in extreme strenuous exercise (more than 8 hours of continuous exercise per week) were also excluded. Participants who exceeded 400 pounds in weight were not included due to the weight limitations of the MRI scanner.

© 2024 Zhang X et al. *JAMA Network Open.*

All procedures complied with institutional guidelines and were approved by the Institutional Review Board at UCLA's Office of Protection for Research Subjects (IRB# 20-002326). All participants provided written informed consent.

Socioeconomic status (SES) was measured using The MacArthur Scale of Subjective Social Status which is a widely used measure of subjective social status [1].

Bioelectrical impedance analysis (BIA) fat mass percentage refers to the percentage of body mass that is composed of fat, as determined through BIA. BIA is a method used to estimate body composition by measuring the impedance (resistance) of electrical flow through the body.

Bioelectrical impedance analysis (BIA) lean body mass percentage refers to the percentage of body mass that consists of lean body tissue, as determined using BIA. In body composition analysis, lean body mass includes everything in the body except for fat mass. This can encompass muscles, bones, organs, and other non-fat tissues.

Diet Questionnaires: All participants completed the UCLA Diet Checklist, which is a questionnaire developed by our institution, intended to represent the diet that best reflects what the participant consumes on a regular basis. The specific diets incorporated into this checklist are summarized in supplemental Table S1. Participants were also allowed to choose "other" if they felt they consumed a diet that was distinct from the ones listed on the Diet Checklist. Our institution's Diet Checklist has been internally validated against the standardized Diet History Questionnaire (DHQ) III. For data analysis, we had two diet categories: the Standard American diet as one category, and all other diets not categorized as Standard American (e.g., Mediterranean, vegan, vegetarian, and gluten-free) were combined as 'Non-Standard American Diet.'

Healthy Eating Index (HEI)-2015[2] was used to access the diet quality. It's a tool designed to evaluate how well a specific set of foods aligns with the Dietary Guidelines for Americans. [3] The components of the HEI-2015 are largely identical to those of the HEI-2010, with the exception that saturated fat and added sugars have replaced empty calories. As a result, the HEI-2015 includes a total of 13 components. HEI-2015 scores can range from 0 to 100, with higher scores indicating better dietary quality.

General Food Cravings Questionnaire – Trait Reduced (G-FCQ-T) [4]: The G-FCQ-T-r is a shorter version of the FCQ-T. This tool is used to assess for a patient's food cravings as stable traits, measuring the features of craving that is consistent over time. It has 15 items: 5 items on the lack of control overeating, 5 items on the thoughts or preoccupation with food, 2 items on intentions and plans to consume food, 2 items on emotions before or during food craving, and 1 item on cues that may trigger food craving. Scores on the FCQ-T are shown to positively associate with eating pathology, BMI, low dieting success, and increase in craving when exposed to food stimuli.

Reward-based Eating Drive (RED) [5]: The RED scale is a 7-item self-report index that looks at three factors: Lack of control over eating, lack of satiation, and preoccupation with food. The scale attempts to understand why some individuals are more vulnerable to persistent weight gain

© 2024 Zhang X et al. *JAMA Network Open.*

than others by delving into the idea that they have a higher drive to eat due to the heightened reward they get by eating.

Three-Factor Eating Questionnaire (TFEQ) [6]: The TFEQ is a 51-item questionnaire which measures three factors of human eating behavior: cognitive restraint of eating (21 items), disinhibition (16 items), and hunger (14 items). Each question is scored either with a 0 or a 1, and the three factors are scored separately, resulting with a final score with three numbers (for example, the maximum possible score is 21-16-14).

Yale Food Addiction Scale (YFAS) [7]: The YFAS is a survey designed to assess whether a patient exhibits signs of addiction towards food, especially those high in food and sugar. It is a 25-item survey, which is based upon the seven substance dependence criteria that are detailed in the DSM-IV, and patients answer questions based on which ones are applicable to them over the past 12 months. Some questions can be answered with a yes/no, while others ask for a frequency (between 'never' to 'four or more times a week or daily'. Food addiction symptoms in our study were measured by the continuous symptom count, reflecting the number of symptoms presented by the respondent.

Connor-Davidson Resilience Scale (CD-RISC) [8] is a 25-item self-rated survey, designed to assess a patient's ability to thrive despite facing adversity. It was created to show that resilience can be quantified, is influenced by health, and can improve after treatment. In this survey, each item is rated 0-4. The higher the overall score, the greater the resilience of the patient.
Spielberger State Trait Anxiety Inventory (STAI)[9] is a 40-item

The Hospital Anxiety and Depression Scale (HAD) [10] is a 14-item questionnaire. The questions are scored on a scale of 0 to 3, corresponding to how much the individual identifies with the question for the past week. It is a well-validated brief inventory for assessment of symptoms of anxiety and depression that has been widely used in studies of medical populations.

Positive Affect Negative Affect Schedule (PANAS) [11] is a psychometric scale developed to measure the largely independent constructs of positive and negative affect, both as states and traits. Positive and negative affect have been shown to relate to other personality states and traits, such as anxiety. Through a factor analysis a list of 10 descriptors for PA scale (attentive, interested, alert, excited, enthusiastic, inspired, proud, determined, strong and active); and 10 descriptors for NA scale (distressed, upset-distressed; hostile, irritable-angry; scared, afraid-fearful; ashamed, guilty; nervous, and jittery) are measured.

Brain Magnetic Resonance Imaging
Whole-brain structural and functional data was acquired using a 3.0T Siemens Prisma MRI scanner (Siemens, Erlangen, Germany). Additional detailed information on the standardized acquisition protocols and quality control measures are provided in previously published studies. [12-19]

Structural MRI Acquisition: High-resolution T1-weighted images were acquired: echo time/ repetition time (TE/TR)=3.26ms/2200ms, field of view=220×220mm, slice thickness=1mm, 176 slices, 256×256 voxel matrix, and voxel size=0.86×0.86×1mm.

© 2024 Zhang X et al. *JAMA Network Open.*

Functional MRI Acquisition: Whole-brain scans were acquired with Participants breathing and blinking normally while watching the slideshow, using an echo planar sequence with the following parameters: TE/TR=28ms/2000ms, flip angle=77º, scan duration=10m6s, FOV=220mm, slices=40, and slice thickness=4.0mm.

Food cue task: Pictures include the following types: unhealthy (high calorie) savory, unhealthy (high calorie) sweet, healthy (low calorie) savory, healthy (low calorie) sweet, and non-food, comprising pixelated images created from food pictures (as a control comparison). All food images were uploaded to E-prime software;[20] half were copied and pixelated to control for color, brightness and contrast. Images were arranged into blocks of 6, comprising either unaltered or pixelated images only, with a total of 18 blocks. Each image was shown for 3 seconds. A black screen with a white crosshair was displayed for 12 seconds before the first block of images, in-between each block of images, and after the final block of images. Two slideshows (order 1 and order 2) were created using the same 18 blocks of images arranged in different orders. Participants watched both sets of images in the scanner.

Brain data preprocessing procedure: The fMRI Expert Analysis Tool (FEAT; version 6.0) included in the FMRIB Software Library (FSL) was used for preprocessing.[21] Preprocessing included motion correction, brain extraction, 100-s high-pass filtering and spatial smoothing with a 5-mm full-width at half-maximum (FWHM) Gaussian kernel. In addition to six motion parameters, nuisance regressors for time points corresponding to motion outliers were included using the FSL motion outliers program (http://fsl.fmrib.ox.ac.uk/fsl/fslwiki/FSLMotionOutliers), which defined outlier time points using the upper threshold of the 75th percentile plus 1.5 times the interquartile range. Functional data were aligned to each participant's structural image, and then registered into Montreal Neurological Institute (MNI) standard space using affine transformation through FSL's Linear Image Registration Tool (FLIRT).

© 2024 Zhang X et al. *JAMA Network Open.*

Results

eTable 1: Diet Checklist Categories

Diet Category	Components
Standard American	High consumption of processed foods, pastas, and breads. Meats, including red meat, fish, eggs, and dairy products consumed.<br>Vegetables and fruits consumed, but not in large quantities
Modified American	High consumption of processed foods, pastas, and breads (mainly whole grain). Poultry, fish, eggs, and dairy products consumed. Red meat consumed in limited quantities<br>Vegetables and fruits consumed, but not in large quantities
Mediterranean	High consumption of fruits, vegetables, bread and other cereals, beans, nuts, and seeds.<br>Olive oil is the key monounsaturated fat source.<br>Dairy products, fish, and poultry are consumed in low to moderate amounts. Little red meat is consumed. Eggs are eaten zero to four times a week and wine is drunk in moderate (or low) amounts.
Paleo	Consumption of basic foods such as plain meat, fish, shellfish, eggs, nuts, vegetables, fruits, berries, and mushrooms.<br>Minimally processed oils, such as avocado, olive or coconut oil, are used for cooking.<br>Dairy products, legumes, dry beans, grains, coffee, alcohol, sugar, and processed foods are excluded.
Vegan	Focus is on plant-based foods. Includes fruits, vegetables, dried beans and peas, grains, seeds, and nuts. Excludes all meat and animal products.
Vegetarian<br>(6 categories)	Focus is on plant-based foods. Includes fruits, vegetables, dried beans and peas, grains, seeds, and nuts.
Vegetarian	Diet excludes all meat but will allow animal-derived ingredients, i.e. honey and gelatin
Lacto-Vegetarian	Diet includes plant foods plus dairy products, no eggs
Ovo-Vegetarian	Diet includes plant foods plus eggs, no dairy
Lacto-Ovo-Vegetarian	Diet includes both dairy products and eggs
Pescatarian	Diet includes fruits, vegetables, dried beans and peas, grains, seeds, and nuts. Excludes all meat except fish.
Raw Vegan/ Raw Food	Consumption of unprocessed vegan foods that have not been heated above 115 degrees Fahrenheit (46 degrees Celsius)
Gluten-Free	Diet includes most foods but avoids the protein, gluten, which is found in wheat, barley, and rye.
Dairy-Free	Diet includes most foods but avoids dairy.
Low FODMAP (Fermentable Oligo-,Di-, Monosaccharides, and Polyols)	Diet limits foods high in sugar and carbohydrates (fructose, lactose, fructans, galactans, and polyols).

© 2024 Zhang X et al. *JAMA Network Open.*

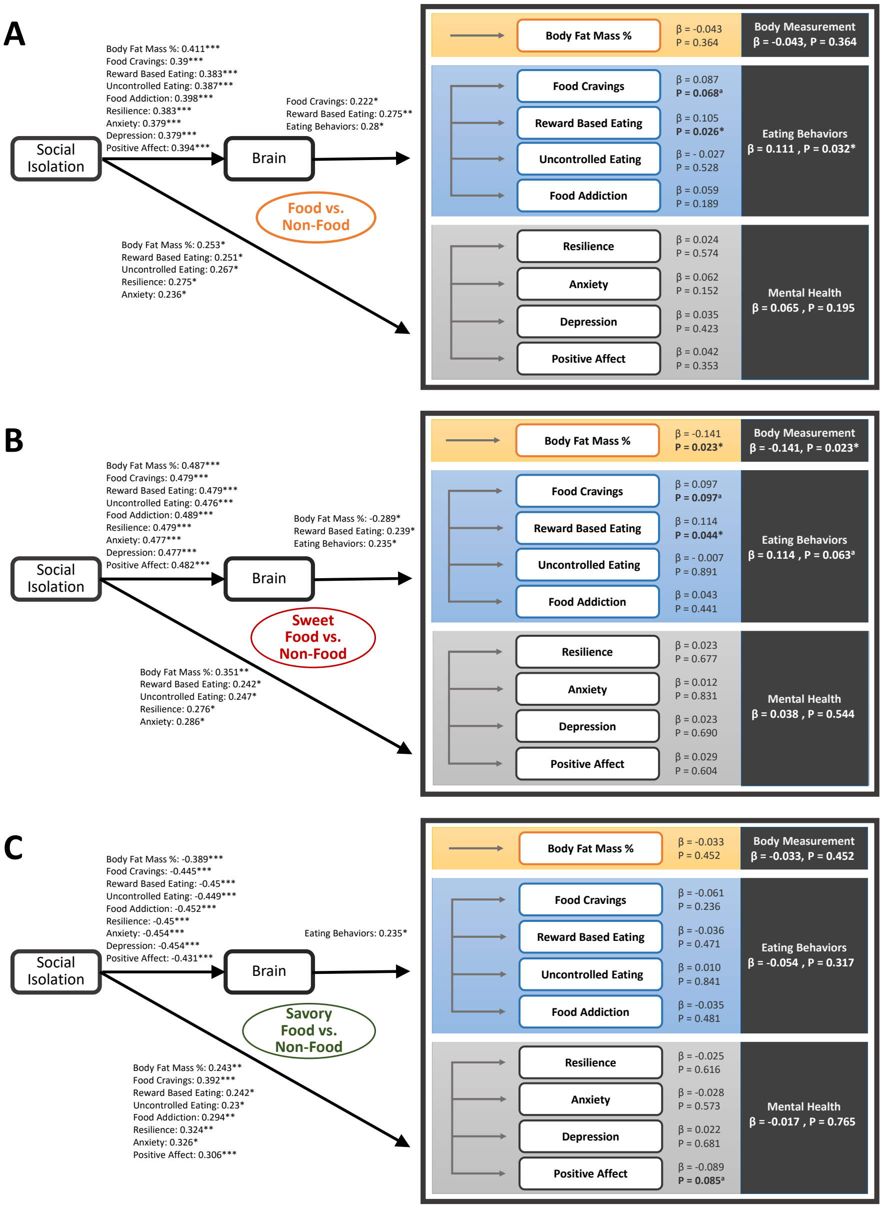

eFigure 2: Mediation Models on Brain Responses to Food Cues Associated With Perceived Isolation and Body Measurement, Eating Behaviors, and Mental Health Factors With Path Coefficients. (A) food vs. non-food; (B) sweet food vs. non-food; and (C) savory food vs. non-food.

Figure Legend: The values adjacent to the arrows connecting brain, clinical/behavioral measures, and perceived isolation represent standardized path coefficients, also known as standardized coefficients. Only significant path coefficients are displayed.

Statistical significance, ^{a} $p < 0.1$, * $p < 0.05$, ** $p < 0.01$, *** $p < 0.001$.

© 2024 Zhang X et al. *JAMA Network Open.*

eReferences

1. Adler NE, Epel ES, Castellazzo G, Ickovics JR. Relationship of subjective and objective social status with psychological and physiological functioning: Preliminary data in healthy, White women. *Health psychology*. 2000;19(6):586.
2. Krebs-Smith SM, Pannucci TE, Subar AF, et al. Update of the healthy eating index: HEI-2015. *Journal of the Academy of Nutrition and Dietetics*. 2018;118(9):1591-1602.
3. You A. Dietary guidelines for Americans. *US department of health and human services and US department of agriculture*. 2015;7
4. Meule A, Teran CB, Berker J, Gründel T, Mayerhofer M, Platte P. On the differentiation between trait and state food craving: Half-year retest-reliability of the Food Cravings Questionnaire-Trait-reduced (FCQ-Tr) and the Food Cravings Questionnaire-State (FCQ-S). *Journal of eating disorders*. 2014;2(1):1-3.
5. Epel ES, Tomiyama AJ, Mason AE, et al. The reward-based eating drive scale: a self-report index of reward-based eating. *PloS one*. 2014;9(6):e101350.
6. Stunkard AJ, Messick S. The three-factor eating questionnaire to measure dietary restraint, disinhibition and hunger. *Journal of psychosomatic research*. 1985;29(1):71-83.
7. Gearhardt AN, Corbin WR, Brownell KD. Preliminary validation of the Yale food addiction scale. *Appetite*. 2009;52(2):430-436.
8. Connor KM, Davidson JR. Development of a new resilience scale: The Connor-Davidson resilience scale (CD-RISC). *Depression and anxiety*. 2003;18(2):76-82.
9. Marteau TM, Bekker H. The development of a six-item short-form of the state scale of the Spielberger State-Trait Anxiety Inventory (STAI). *Br J Clin Psychol*. Sep 1992;31(3):301-6. doi:10.1111/j.2044-8260.1992.tb00997.x
10. Zigmond AS, Snaith RP. The hospital anxiety and depression scale. *Acta Psychiatr Scand*. Jun 1983;67(6):361-70. doi:10.1111/j.1600-0447.1983.tb09716.x
11. Watson D, Clark LA, Tellegen A. Development and validation of brief measures of positive and negative affect: the PANAS scales. *Journal of personality and social psychology*. 1988;54(6):1063.
12. Osadchiy V, Mayer EA, Gao K, et al. Analysis of brain networks and fecal metabolites reveals brain-gut alterations in premenopausal females with irritable bowel syndrome. *Transl Psychiatry*. Nov 2 2020;10(1):367. doi:10.1038/s41398-020-01071-2
13. Osadchiy V, Mayer EA, Bhatt R, et al. History of early life adversity is associated with increased food addiction and sex-specific alterations in reward network connectivity in obesity. *Obes Sci Pract*. Oct 2019;5(5):416-436. doi:10.1002/osp4.362
14. Labus JS, Van Horn JD, Gupta A, et al. Multivariate morphological brain signatures predict patients with chronic abdominal pain from healthy control subjects. *Pain*. Aug 2015;156(8):1545-1554. doi:10.1097/j.pain.0000000000000196
15. Gupta A, Mayer EA, Labus JS, et al. Sex Commonalities and Differences in Obesity-Related Alterations in Intrinsic Brain Activity and Connectivity. *Obesity (Silver Spring)*. Feb 2018;26(2):340-350. doi:10.1002/oby.22060
16. Gupta A, Mayer EA, Hamadani K, et al. Sex differences in the influence of body mass index on anatomical architecture of brain networks. *Int J Obes (Lond)*. Aug 2017;41(8):1185-1195. doi:10.1038/ijo.2017.86

© 2024 Zhang X et al. *JAMA Network Open.*

17. Gupta A, Mayer EA, Acosta JR, et al. Early adverse life events are associated with altered brain network architecture in a sex- dependent manner. *Neurobiol Stress*. Dec 2017;7:16-26. doi:10.1016/j.ynstr.2017.02.003
18. Dong TS, Mayer EA, Osadchiy V, et al. A Distinct Brain-Gut-Microbiome Profile Exists for Females with Obesity and Food Addiction. *Obesity (Silver Spring)*. Aug 2020;28(8):1477-1486. doi:10.1002/oby.22870
19. Dong TS, Gupta A, Jacobs JP, et al. Improvement in Uncontrolled Eating Behavior after Laparoscopic Sleeve Gastrectomy Is Associated with Alterations in the Brain-Gut-Microbiome Axis in Obese Women. *Nutrients*. Sep 24 2020;12(10)doi:10.3390/nu12102924
20. Schneider W, Eschman A, Zuccolotto A. *E-Prime: User's guide. Reference guide. Getting started guide*. Psychology Software Tools, Incorporated; 2002.
21. Smith SM, Jenkinson M, Woolrich MW, et al. Advances in functional and structural MR image analysis and implementation as FSL. *Neuroimage*. 2004;23:S208-S219.

© 2024 Zhang X et al. *JAMA Network Open.*
