# Supplementary material for: Social Isolation, Brain Food Cue Processing, Eating Behaviors, and Mental Health Symptoms
Source: JAMA Netw Open. 2024 Apr 4;7(4):e244855. doi: 10.1001/jamanetworkopen.2024.4855 (PMC11192185; doi:10.1001/jamanetworkopen.2024.4855)
Supplement: Supplement 2. — Data Sharing Statement [file jamanetwopen-e244855-s002.pdf]

Data Sharing Statement

Zhang. Social Isolation, Brain Food Cue Processing, Eating Behaviors, and Mental Health Symptoms. *JAMA Netw Open*. Published April 04, 2024.
doi:10.1001/jamanetworkopen.2024.4855

Data
Data available: Yes
Data types: Data dictionary, Deidentified participant data
How to access data: Deidentified individual participant data can be shared upon request and will be made available through the Center's pain repository portal (https://www.painrepository.org/). To access the data, participants will fill out a user agreement, upon which access to the data will be made available through a secure password protected portal.
When available: With publication

Supporting Documents
Document types: None

Additional Information
Who can access the data: researchers whose proposed use of the data has been approved
Types of analyses: for a specified purpose
Mechanisms of data availability: with a signed data access agreement
